# Handgrip strength as a moderator of the influence of age on olfactory impairment in US adult population ≥ 40 years of age

**DOI:** 10.1038/s41598-021-93355-w

**Published:** 2021-07-08

**Authors:** Robinson Ramírez-Vélez, José Francisco López-Gil, Mikel López Sáez de Asteasu, Mikel Izquierdo, Antonio García-Hermoso

**Affiliations:** 1 Navarrabiomed, Complejo Hospitalario de Navarra (CHN), Universidad Pública de Navarra (UPNA), IdiSNA, Pamplona, Spain; 2 CIBER of Frailty and Healthy Aging (CIBERFES), Instituto de Salud Carlos III, Madrid, Spain; 3 Departamento de Actividad Física y Deporte, Facultad de Ciencias del Deporte, Universidad de Murcia, Murcia, Spain; 4 Universidad de Santiago de Chile (USACH), Escuela de Ciencias de la Actividad Física, el Deporte y la Salud, Santiago, Chile

**Keywords:** Population screening, Disease prevention

## Abstract

The aim of this study was to determine whether handgrip strength attenuates the negative relationship between age and olfactory function in a representative US population sample 40 years old and over. A cross-sectional study was performed with 2861 adults from the US National Health and Nutrition Examination Survey NHANES (2013–2014). An 8-item odor identification test was applied to determine olfactory function. Muscle strength was determined through a handgrip dynamometer (defined as the sum of the largest handgrip strength reading from right and left hands). Moderation analysis was performed to test whether the association between age and olfactory impairment was moderated by handgrip strength. Moderation analysis highlighted two regions of significance: the first region was found at < 56.6 kg, indicating that the adverse influence of age on olfactory function may be greater for the participants in this area; the second region was found at ≥ 56.6 kg, indicating that the negative impact of age on olfactory function disappeared for adults who were above this estimate point. In conclusion, handgrip strength, a general indicator of muscle strength, moderates the relationship between age and olfactory ability in a US adult population aged 40 years and older. Our findings are clinically relevant, since they emphasize the importance of muscular fitness in adulthood and old age by diminishing the deleterious effect of aging on olfactory performance.

## Introduction

Olfactory impairment, characterized by increased odor thresholds and weakened odor discrimination and recognition^1^ in community-dwelling older people is an early sign of geriatric syndromes, including disability and reduced quality of life^2^, depressive symptoms^3^, subjective memory impairment^4^, Alzheimer disease^5^, sarcopenia/frailty^6^ and preclinical dementia^7^. The US National Health and Nutrition Examination Survey (NHANES), a nationally representative multistage probability survey among the US population, included a chemosensory test in the 2011–2014 protocol. In an analysis of the first-year (2012) results of people aged 40 years and older, it was reported olfactory impairment in 12.4% of participants (13.3 million adults; 55% men/45% women), including 3.2% anosmic/severe hyposmic (3.4 million; 74% men/26% women)^8^. Similarly, using a larger sample size (NHANES, 2013–2014), another study found that smell and taste dysfunction affected ∼ 20.5 million (13.5%) and 26.3 million (17.3%) individuals, respectively^9^.

Less is known about the specific relationship between sensory impairment, muscle weakness, and low muscle mass in adults. Handgrip strength is an objective measure of upper body muscle strength and is considered as a general indicator of muscle strength^10,11^. This measure is related to a broad range of clinically meaningful health outcomes in older adults^12^. A recent observational study found that older adults (mean age 73 years) with muscle weakness (i.e., sarcopenia/frailty phenotype) had decreased olfactory acuity^6^. Furthermore, sarcopenia and frailty are often caused by anorexia related to olfactory and gustatory impairment^13^. Loss of muscular fitness, an age-related condition of increased vulnerability, is associated with a higher risk of several adverse outcomes^14^, including mild cognitive impairment, Alzheimer disease, Parkinson disease and mortality^15^. Loss of olfactory capacity might be an early sign of associated age-related changes in muscle strength, before muscle mass decline is apparent^16^. In this line, a relationship between sensory loss (e.g., combinations of smell, hearing and vision) and handgrip strength has recently been reported among women^17^.

To the best our knowledge, the influence of handgrip strength in the relation between age and olfactory capacity remains unknown. As mentioned in the literature review, there is a growing body of literature that recognizes weak handgrip strength as predictive value for adverse outcomes^18,19^. Therefore, one might speculate that physical activity and improvements in muscle strength might help to prevent olfactory decline^20^. Based on the above, our hypothesis is that sufficient levels of muscle strength in adulthood and old age could be associated with lower sensory deterioration of smell over the years. Thus, the aim of this study was to determine whether combined handgrip strength (defined as the sum of the largest grip strength reading from right and left hands) attenuates the relationship between age and olfactory impairment in a nationally representative sample of older adults.

## Methods

### Study design and sample population

NHANES 2013–2014 is a cross-sectional US survey designed to assess the health and nutritional status of adults and children. This survey employed a computer-assisted personal interview system. The olfactory examination is a relatively recent component performed in participants aged 40 years and older. Women who were pregnant (had either self-reported pregnancy or positive urine pregnancy test) or breastfeeding were excluded from the olfactory test. Among the 6467 representative individuals aged ≥ 40 years who were eligible for the survey, 3708 (57.3%) were interviewed and 847 participants were removed because of missing values (handgrip strength, smell test, potential covariates, etc.). Thus, data from 2861 (44.2% attrition rate) remaining participants were included in the final analysis.

All procedures were performed according to the ethical standards of the institutional and/or national research committee (National Health and Nutrition Examination Survey, NCHS IRB/ERB Protocol Number: NHANES 2013–2014, Continuation of Protocol #2011-17). No additional consent was required for our study, as the information analyzed did not contain personal identifiers. The information was acquired through a simple stratified multi-stage probability sampling of non-institutionalized civilian citizens of the US.

### Anthropometric measurements

Participants’ height and weight were determined with a stadiometer and a digital weight scale, respectively. Body mass index (BMI) was computed by dividing weight (kg) by height (m^2^). Participants were categorized based on BMI group as 18 to < 25 kg/m^2^ (normal weight), BMI 25 to < 30 kg/m^2^ (overweight), and BMI ≥ 30 kg/m^2^ (obese)^21^.

### The 8-item odor identification test

The two four-item versions (A and B) of the NHANES Pocket Smell Test (Sensonics International, Haddon Heights, NJ, USA), were consecutively developed into an 8-item ‘scratch and sniff’ test^22^. Prior to the olfactory test, a series of pre-exam screening questions were carried out to determine any conditions that could potentially bias the test results, such as, runny nose, sinus pain, or nasal blockage. The eight odorants (a-chocolate, b-strawberry, c-smoke, d-leather, e-soap, f-grape, g-onion, and h-natural gas) were presented in a fixed order. Participants were required, in a forced-choice situation, to ascertain each odorant from four possible names. A recent validation study demonstrated moderate-to-good test–retest reliability of the NHANES smell protocol (intraclass correlations were 0.82 and 0.69 for 2-week and 6-month intervals, respectively)^22^. Olfactory impairment was considered when the participant was unable to identify at least 6 of 8 odorants^23^.

### Upper muscle strength test

Muscle strength was determined through a handgrip test using a handgrip dynamometer (T.K.K. 5401, Grip-D, Takei, Japan), adjusting for differences in hand size for each participant. A trained examiner explained and demonstrated the protocol. Participants were instructed to maintain a proper stance, standing with their feet hip-width apart and even, toes pointing forward, knees comfortable but not bent, shoulders back and chest up, eyes straight ahead, shoulder abducted ∼ 10°, arm straight down side, elbow fully extended, and wrist in neutral position. The test was repeated three times per hand, interchanging hands with a 60-s rest between measurements on the same hand. Combined handgrip strength was the sum of the largest handgrip strength reading from each hand.

### Covariates

The covariates integrated in the adjusted analyses were according to a conceptual model based on the scientific literature^9^. The in-house questionnaire collected data on race/ethnicity (non-Hispanic Black, other Hispanic, non-Hispanic White, Mexican American and other race), sex, education level (some college or AA degree, college graduate or above, high-school graduate, 9–11th grade, and less than 9th grade), ratio of family income to federal poverty threshold, marital status (living with partner married, never married, separated, divorced and widowed), BMI, smoking status (< 100 cigarettes in life and ≥ 100 cigarettes in life), alcohol consumption (< 12 alcohol drinks per year and ≥ 12 alcohol drinks per year), dietary intake and self-reported chronic diseases (stroke, cancer, asthma, cardiovascular disease, diabetes and hypertension).

### Statistical analysis

Descriptive data are expressed as means and standard deviation (SD) for continuous variables, and numbers and percentages for categorical variables. Pearson’s chi-square test and Student’s t test were used to compare categorical and continuous variables, respectively. Linear regression analysis was performed to evaluate the associations of absolute handgrip strength with the objective olfactory test score and age. Prior analysis indicated no interaction between sex and the 8-item odor identification test score (*p* = 0.289). Also, a recent meta-analysis demonstrated that although there exists certain sex differences in olfactory performance, the effects are notably small, and they translate to very low absolute differences in olfactory test performance^24^. For these reasons, both sexes were included together in order to increase statistical power.

Analyses of covariance (ANCOVA) were applied to test the differences between means according to each result on the 8-item odor identification test. All assumptions were checked beforehand (i.e., normality and homoscedasticity).

Moderation analysis was performed using the PROCESS macro 3.5 in SPSS (IBM SPSS Statistics for Windows, Version 25.0, Armonk, NY, USA). The PROCESS macro uses ordinary least squares analysis to predict continuous variables (absolute handgrip strength) and with bootstrapping (10,000 bootstrapped samples) to determine the moderated influences^25^. The Johnson-Neyman approach was applied to test statistically-significant interactions and identify regions of significance, and three different points of the moderator were established: high (+ 1 SD), average (mean) and low (− 1 SD). We used this procedure to obtain a threshold of statistical significance from the conditional model. All the analyses were adjusted for sex, race/ethnicity, ratio of family income to federal poverty threshold, marital status, education level, BMI, dietary intake, smoking status, alcohol consumption and self-reported chronic diseases. Our a priori plan was to use an alpha level of 0.05. However, we finally considered a p-value lower than 0.005 as statically significant in order to control for type 1 error^26^.

## Results

Table 1 shows the characteristics of the study participants. The mean (± SD) age of participants with olfactory impairment (failing to identify at least 6/8 odorants) was significantly higher (65.3 ± 11.9 years) than those without impairment (57.6 ± 11.4 years). Also, absolute handgrip strength levels were lower in participants with olfactory impairment than in those without (31.96 ± 10.64 vs 34.38 ± 10.73 kg; *p* < 0.001).

**Table 1:** Characteristics of participants.

Variables	Smell impairment		
	No (n = 2403; 84.0%)	Yes (n = 457; 16.0%)	*p*
	M (SD)/n (%)	M (SD)/n (%)	
**Sociodemographic**			
Age, y	57.6 (11.4)	65.3 (11.9)	< 0.001
Men, %	1292 (53.8)	182 (39.9)	< 0.001
Ratio family income to poverty	2.58 (1.76)	2.22 (1.59)	< 0.001
Race/ethnicity, % non-Hispanic White	1164 (48.4)	184 (40.3)	0.011
Marital status, % married	1442 (60.0)	265 (58.0)	< 0.001
Education level, % AA grade	749 (31.2)	102 (22.3)	< 0.001
**Anthropometric measurements**			
Weight, kg	83.11 (21.33)	80.68 (20.80)	0.025
Height, cm	167.0 (10.0)	166.3 (10.4)	0.071
BMI, kg/m^2^	29.72 (6.93)	29.13 (6.79)	0.092
Overweight/obese, %^a^	1805 (75.1)	337 (73.7)	0.535
**Muscular fitness**			
Combined handgrip strength, kg	34.38 (10.73)	31.96 (10.64)	< 0.001
**Dietary intake**			
Total energy, kcal	1995.0 (786.7)	1847.2 (799.7)	< 0.001
Carbohydrates, g	237.4 (101.6)	227.5 (101.2)	0.057
Proteins, g	79.9 (34.3)	74.5 (38.7)	0.002
Fats, g	77.4 (36.8)	68.7 (36.3)	< 0.001
**Lifestyle**			
Alcohol consumption^b^, % yes	1784 (74.2)	296 (64.8)	< 0.001
Smoking^c^, % yes	1103 (45.9)	241 (52.7)	0.007
**Comorbidities**			
Asthma, % yes	13 (0.5)	2 (0.4)	1.000
Stroke, % yes	86 (3.6)	45 (9.8)	< 0.001
Cancer, % yes	361 (12.5)	113 (17.7)	0.008
Hypertension, % yes	1180 (49.1)	240 (52.5)	0.181
Diabetes, % yes	409 (17.0)	103 (22.5)	0.006
Hearth disease, % yes	2131 (88.7)	405 (88.6)	0.971
**Odor identification**			
Chocolate, % yes	2125 (88.4)	267 (58.4)	< 0.001
Strawberry, % yes	2076 (86.4)	215 (47.0)	< 0.001
Smoke, % yes	2280 (94.9)	234 (51.2)	< 0.001
Leather, % yes	2061 (85.8)	163 (35.7)	< 0.001
Soap, % yes	2331 (97.0)	343 (75.1)	< 0.001
Grape, % yes	1733 (72.1)	98 (21.4)	< 0.001
Onion, % yes	2370 (98.6)	349 (76.4)	< 0.001
Natural gas, % yes	2256 (93.9)	258 (56.5)	< 0.001

Data presented as mean (standard deviation) or numbers (percentages).

^a^Nutritional status was defined following the Expert Panel on the Identification, Evaluation, and Treatment of Overweight in Adults criteria^21^.

^b^Alcohol consumption was determined by the question “Had at least 12 alcohol drinks per year?”.

^c^Smoking was determined by the question “Smoked at least 100 cigarettes in life?”.

The associations between both age and objective olfactory test score and absolute handgrip strength are shown in Table 2. After adjustment for several covariates, we found a positive association between handgrip strength and objective olfactory test score (*β* = 0.131; [95% CI 0.831–1.221]; *p* < 0.001). Conversely, we found a negative association between absolute handgrip strength and age (*β* =  − 0.343; [95% CI − 0.332 to − 0.290]; *p* < 0.001).

**Table 2:** Association between age and 8-item odor identification test score and absolute handgrip strength.

Predictors	B	SE	*β*	*p*	LLCI	ULCI
**Unadjusted model**						
Age	− 0.329	0.014	− 0.369	< 0.001	− 0.358	− 0.301
8-Item odor identification test	0.749	0.134	0.098	< 0.001	0.487	1.012
**Adjusted model**						
Age	− 0.311	0.011	− 0.343	< 0.001	− 0.332	− 0.290
8-Item odor identification test	1.026	0.099	0.131	< 0.001	0.831	1.221

Adjusted model: sex, race/ethnicity, ratio family income to poverty, marital status, education level, body mass index, dietary intake, smoking status, alcohol consumption and self-reported chronic diseases.

**Figure 1:**
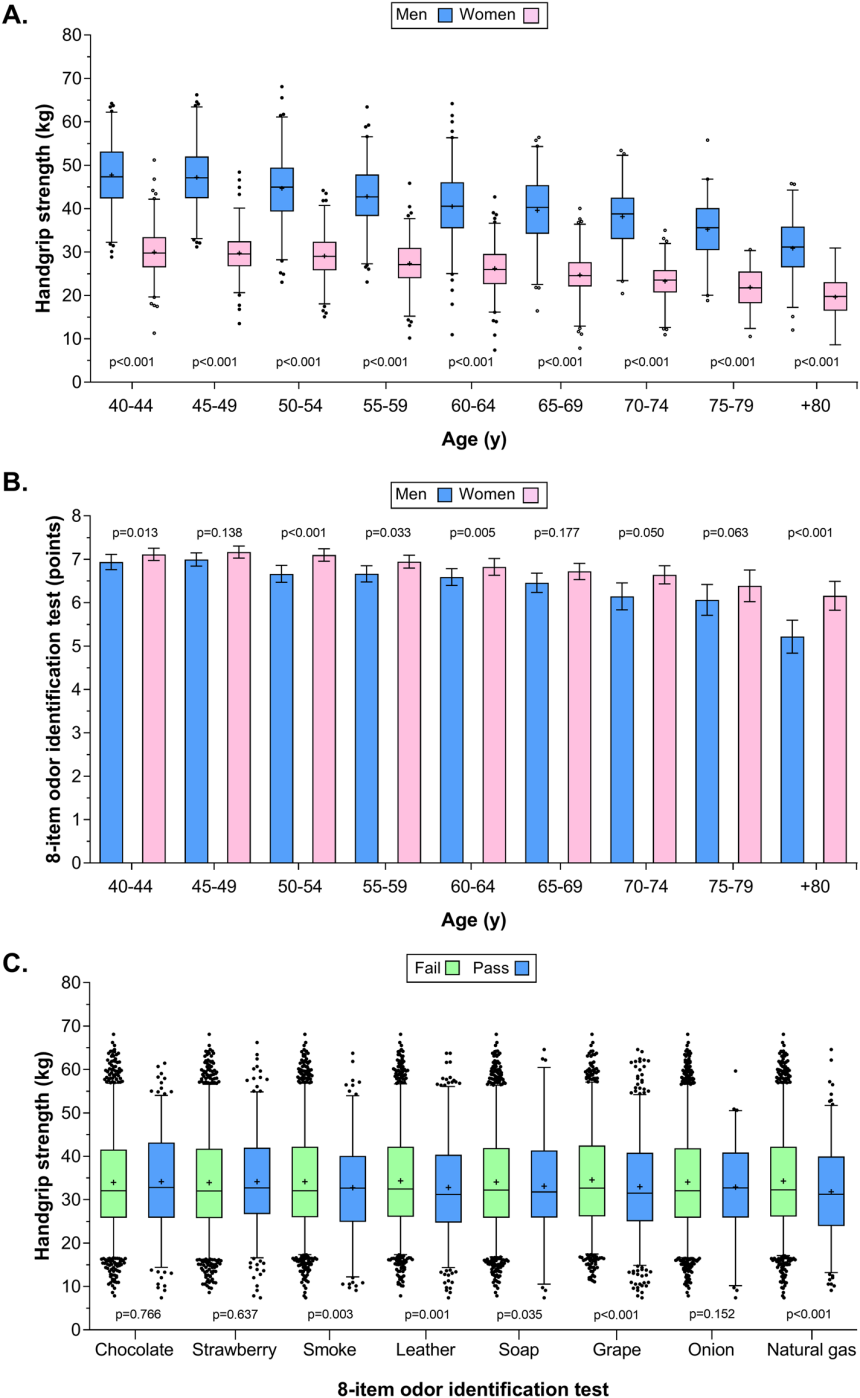
Absolute handgrip strength and odor identification results. (**A**) Differences between mean absolute handgrip strength by sex and age group. (**B**) Differences between mean absolute handgrip strength and result of the 8-item odor identification test. (**C**) Differences between mean absolute handgrip strength and result of the 8- item odor identification test (test fail or pass). In the box and whiskers, the + indicates the average value. The error band indicates the 2.5–97.5 percentile for each value. Outliers are plotted as individual points.

Overall, men showed higher handgrip strength than women (41.8 kg vs. 26.6 kg) across all age groups (*p* < 0.001) (Fig. 1A). In both sexes, the highest handgrip strength values were observed in the age groups of 40–44 and 45–49 years (men, 47.2 kg; women, 29.8 kg, *p* < 0.001), which subsequently declined in later years. Figure 1B shows the correct answer average for the 8-item odor test according to the age and sex groups. The mean of the objective olfactory test score was lower among men and older age groups (*p* < 0.001). As shown in Fig. 1C, higher handgrip strength was related to lower errors (i.e., test passes) on smoke, leather, grape, and natural gas odors (*p* < 0.005).

**Figure 2:**
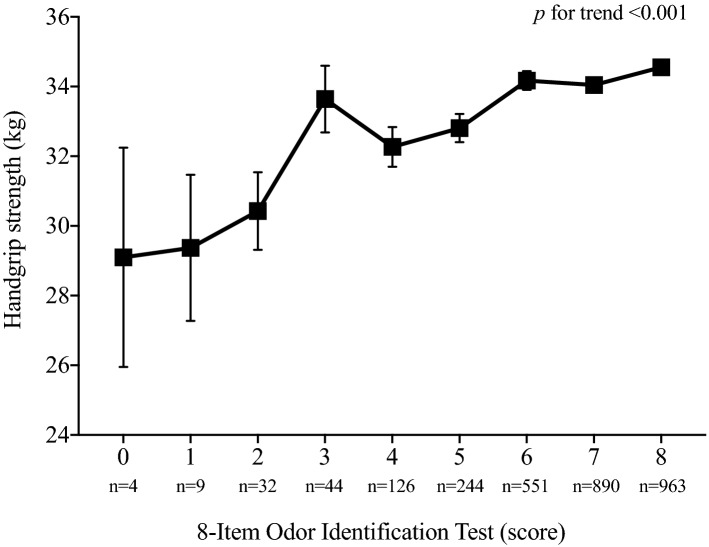
Differences between mean absolute handgrip strength according to the number of positive identifications on the 8-item odor identification test. Adjusted by sex, race/ethnicity, ratio family income to poverty, marital status, education level, dietary intake, smoking status, alcohol consumption and self-reported chronic diseases.

Differences between mean absolute handgrip strength according to the number of positive identifications on the 8-item odor identification test is shows in Fig. 2. These results revealed statistically significant differences between the number of correct identifications in the olfactory test and the absolute handgrip strength (*p* for trend < 0.001) after adjusting for several covariates (Fig. 2).

**Figure 3:**
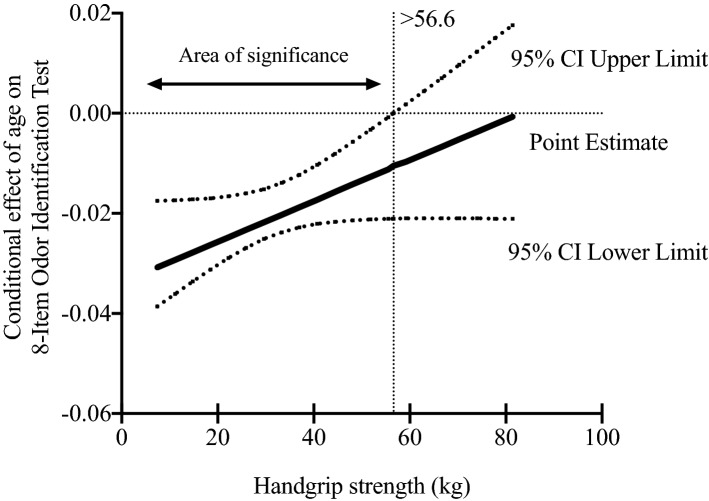
Moderation analysis using the Johnson-Neyman technique. Moderating effect of handgrip strength on the relation between age and olfactory function. Data are presented as a regression point estimate (beta unstandardized) and 95% confidence intervals. Adjusted by sex, race/ethnicity, ratio family income to poverty, marital status, education level, dietary intake, smoking status, alcohol consumption and self-reported chronic diseases.

Lastly, we explored the moderating effect that handgrip strength has on the relation between age and olfactory function. Figure 3 shows the conditional effect of age on olfactory function. The Johnson-Neyman technique revealed the different moderator values (absolute handgrip strength) by means of the slope and the different regions of statistical significance. The first region is shown at < 56.6 kg, indicating that the adverse influence of age on olfactory function may be greater for the participants in this region. The second region was found at ≥ 56.6 kg, indicating that the negative impact of age on olfactory function disappeared in participants who were above this estimate point.

## Discussion

The present study investigates whether the association between age and olfactory impairment in a nationally representative sample of US adults 40 years or older is moderated by absolute handgrip strength. Confirming previous studies^8,9^, our results reveal a positive association between age and olfactory impairment. We also show that this association is moderated by handgrip strength, an assessment which can represent general muscle strength.

Depending on study methodology and sample characteristics, olfactory impairment has been estimated to affect 40–70% of the general aging population^23^, as opposed to 5–15% in younger age groups^27^. In the present study, 18.1% of adults aged ≥ 40 years had olfactory impairment, which is slightly lower than the prevalence reported by other authors (19.1%) in a Swedish population-based study (n = 1387, aged 20–85 years or more)^28^. Likewise, in a population-based survey from Germany (n = 1312, age range 25–75 years) the prevalence of olfactory dysfunction (questionnaire-based self-assessment or psychophysical olfactory tests) was estimated as 21.6%^29^. The relatively high prevalence of olfactory impairment in adults aged ≥ 40 years is clinically relevant and a major public health concern because it remains underdiagnosed, and is associated with it increases the risk of age-related neurodegenerative diseases, such as Alzheimer and Parkinson^30^ as well as psychological issues including depression, anxiety, and other negative emotions^31^.

Human olfactory neuroepithelium has self-renewal capacity, and the balance between olfactory neurogenesis and cell death is responsible for the maintenance of an adequate number of olfactory receptor neurons^32^. In the context of aging and olfactory function, age is associated with retarded receptor cell regeneration, for example, a reduction in the ratio of living to dead or dying receptor cells has been reported in aging rats^33^. Similarly, the restoration of chemically damaged olfactory epithelium in older mice is slow or nonexistent^34^.

Muscle deterioration in old age is primarily explained by neural and muscular decline due to the aging process, and concomitant physical inactivity and malnutrition^35^. In the same line, olfactory dysfunction is known to be linked to poor general health, suggesting that risk factors may accumulate to exacerbate age-related olfactory losses^36^. The mechanisms by which factors interact (i.e., change in body composition and/or muscular decline) to promote olfactory dysfunction in individuals free from neurodegenerative disease remain, however, unclear. A novel finding of our study is that handgrip strength may diminish the negative influence of aging on olfactory function. A plausible mechanism that might explain this association could be that intrinsic muscle capacity is linked to increases in the availability of growth factors that, in turn, enhance cognitive function, cell plasticity, and neurogenesis^37^. “As adequate protein intake is crucial to minimize age-related loss of muscle mass/strength, there is an obvious relationship between protein consumption, olfactory function and muscle strength^38^. A reduction in olfactory function may impact food selection and trigger appetite loss, weight/muscle loss, and promote malnutrition^39^, and there is considerable evidence to support the role of protein intake as an anabolic stimulus for muscle protein synthesis to prevent and manage sarcopenia^40^. In this sense, we performed our analyses adjusting for total energy intake, carbohydrates, proteins and fats.

Weak handgrip strength is indicative of decline in upper extremity strength with high predictive value for adverse outcomes^18,19^. Accordingly, physical activity that improves muscle strength might help to prevent olfactory decline^20^. Identifying factors that can exacerbate or mitigate olfactory dysfunction, including comorbidities or lifestyle, could be crucial in establishing public awareness campaigns on related harmful effects^41^. In this line, our findings point out the importance of establishing sufficient levels of muscle strength in adulthood and old age, to prevent sensory deterioration of smell over the years. Supporting this notion, regular exercise was reported to be associated with a lower 10-year cumulative incidence of olfactory impairment^42^. Also, as olfactory loss represents a risk factor for weight loss, aerobic exercise might preserve olfactory function in selected populations, such as patients with Parkinson disease^43,44^. These studies provide rationale to investigate the idea that exercise and having higher levels of fitness (e.g., muscle strength) may facilitate neuroplasticity of the olfaction system. We speculate that a possible explanation could lie in the relationship between muscle strength and cognitive function^45^, as olfactory function has been linked in a similar way to cognitive function^46^. Moreover, if exercise is suggested to prevent or delay olfactory deterioration in adults, the type of physical exercise (i.e., aerobic, strength or combined training) should be considered. However, the underlying mechanisms await further investigation.

The main strength of the present study is that includes a nationally representative sample of adults and elderly in the US population. In addition, detailed demographical, lifestyle, nutritional, and medical information are available in the questionnaire and laboratory datasets. Most previous studies that explored the association between olfactory impairment and aging were performed in older age groups^23,47–49^. In this sense, although a recent short communication reported that normal levels of handgrip strength were associated with less likelihood of olfactory impairment in an older US population (aged 60 or older)^47^, our study extends this analysis and determines how handgrip strength can moderate the relationship between aging and smell impairment. We also included all adult subjects (> 40 years) participating in NHANES. Another strength of our study is that, in contrast to the aforementioned communication, our analyses were adjusted for important covariates including a wide spectrum of comorbidities or dietary intake, as it has demonstrated that chemosensory disturbances are influenced by a range of demographic and health factors^9^. Finally, the handgrip test is an objective method commonly used for measuring the overall “proxy” muscle strength; thus, it is an easy test to implement to study the potential for olfactory impairment.

The present study is not without limitations. Firstly, due to the cross-sectional nature of the study, the ability to establish causal relationships is limited. Secondly, smell function was determined at a single moment in time, which could not be extrapolated of to a longer time-frame. However, the smell measurements applied in the study have been validated as highly reproducible over a 6-month period^22^. Third, odor identification test could indirectly measure cognitive and verbal functioning^50^, rather than physiological factors affecting smell. Therefore, it may be more appropriate to use the olfactory threshold and discrimination as the dependent variable in future research. Lastly, although we controlled for a number of covariates in our ANCOVA models, some other potential confounders may also exist and bias the results, such as cognitive function (which was not assessed in this sub-analysis).

To conclude, our research indicates that handgrip strength, a general indicator of muscle strength, moderates the relationship between age and olfactory performance in a nationally representative population of US adults aged 40 years old or older. Our findings are clinically relevant because aging is the most frequent cause of olfactory impairment. It might be possible that screening for handgrip strength and emphasizing the importance of physical fitness in adulthood and old age can help to diminish the deleterious effect of aging on smell impairment. However, more studies with other study designs are required in order to test for causal relationships﻿.

## References

[1] Doty RL, Kamath V (2014). The influences of age on olfaction: A review. Front. Psychol..

[2] Miwa T, Furukawa M, Tsukatani T, Costanzo RM, DiNardo LJ, Reiter ER (2001). Impact of olfactory impairment on quality of life and disability. Arch. Otolaryngol. Head Neck Surg..

[3] Boesveldt S, Lindau ST, McClintock MK, Hummel T, Lundstrom JN, Lindstrom JN (2011). Gustatory and olfactory dysfunction in older adults: A national probability study. Rhinology.

[4] Sohrabi HR, Bates KA, Rodrigues M, Taddei K, Laws SM, Lautenschlager NT, Dhaliwal SS, Johnston ANB, Mackay-Sim A, Gandy S, Foster JK, Martins RN (2009). Olfactory dysfunction is associated with subjective memory complaints in community-dwelling elderly individuals. JAD.

[5] Conti MZ, Vicini-Chilovi B, Riva M, Zanetti M, Liberini P, Padovani A, Rozzini L (2013). Odor identification deficit predicts clinical conversion from mild cognitive impairment to dementia due to Alzheimer’s disease. Arch. Clin. Neuropsychol..

[6] Harita M, Miwa T, Shiga H, Yamada K, Sugiyama E, Okabe Y, Miyake Y, Okuno T, Iritani O, Morimoto S (2019). Association of olfactory impairment with indexes of sarcopenia and frailty in community-dwelling older adults. Geriatr. Gerontol. Int..

[7] Lafaille-Magnan M-E, Poirier J, Etienne P, Tremblay-Mercier J, Frenette J, Rosa-Neto P, Breitner JCS, For the PREVENT-AD Research Group (2017). Odor identification as a biomarker of preclinical AD in older adults at risk. Neurology.

[8] Hoffman HJ, Rawal S, Li C-M, Duffy VB (2016). New chemosensory component in the US National Health and Nutrition Examination Survey (NHANES): First-year results for measured olfactory dysfunction. Rev. Endocr. Metab. Disord..

[9] Liu G, Zong G, Doty RL, Sun Q (2016). Prevalence and risk factors of taste and smell impairment in a nationwide representative sample of the US population: A cross-sectional study. BMJ Open.

[10] Porto JM, Nakaishi APM, Cangussu-Oliveira LM, Freire Júnior RC, Spilla SB, de Abreu DCC (2019). Relationship between grip strength and global muscle strength in community-dwelling older people. Arch. Gerontol. Geriatr..

[11] Wind AE, Takken T, Helders PJM, Engelbert RHH (2010). Is grip strength a predictor for total muscle strength in healthy children, adolescents, and young adults?. Eur. J. Pediatr..

[12] McGrath RP, Kraemer WJ, Snih SA, Peterson MD (2018). Handgrip strength and health in aging adults. Sports Med..

[13] Visvanathan R (2015). Anorexia of aging. Clin. Geriatr. Med..

[14] Soysal P, Hurst C, Demurtas J, Firth J, Howden R, Yang L, Tully MA, Koyanagi A, Ilie PC, López-Sánchez GF, Schwingshackl L, Veronese N, Smith L (2020). Handgrip strength and health outcomes: Umbrella review of systematic reviews with meta-analyses of observational studies. J. Sport Health Sci..

[15] García-Hermoso A, Cavero-Redondo I, Ramírez-Vélez R, Ruiz JR, Ortega FB, Lee D-C, Martínez-Vizcaíno V (2018). Muscular strength as a predictor of all-cause mortality in an apparently healthy population: A systematic review and meta-analysis of data from approximately 2 million men and women. Arch. Phys. Med. Rehabil..

[16] Katsimpris A, Jürgens C, Lüdtke L, Bahls M, Ittermann T, Gläser S, Dörr M, Ewert R, Volaklis K, Felix SB, Tost F, Völzke H, Meisinger C, Baumeister SE (2020). Association between cardiorespiratory fitness and handgrip strength with age-related macular degeneration: A population-based study. Br. J. Ophthalmol..

[17] Gopinath B, Liew G, Burlutsky G, Mitchell P (2019). Associations between vision, hearing, and olfactory impairment with handgrip strength. J. Aging Health.

[18] Ramírez-Vélez R, Correa-Bautista JE, García-Hermoso A, Cano CA, Izquierdo M (2019). Reference values for handgrip strength and their association with intrinsic capacity domains among older adults. J. Cachexia. Sarcopenia Muscle.

[19] Ramírez-Vélez R, Pérez-Sousa MÁ, Cano-Gutierrez CA, Izquierdo M, García-Hermoso A, Correa-Rodríguez M (2020). Association between ideal cardiovascular health score and relative handgrip strength of community-dwelling older adults in Colombia. J. Am. Med. Dir. Assoc..

[20] Zhang C, Li D, Wang X (2020). Role of physical exercise type in olfactory deterioration in ageing. Rhinology.

[21] Clinical guidelines on the identification, evaluation, and treatment of overweight and obesity in adults: executive summary: Expert Panel on the Identification, Evaluation, and Treatment of Overweight in Adults. *Am. J. Clin. Nutr.* **68**, 899–917. 10.1093/ajcn/68.4.899 (1998). 10.1093/ajcn/68.4.899 9771869

[22] Rawal S, Hoffman HJ, Honda M, Huedo-Medin TB, Duffy VB (2015). The taste and smell protocol in the 2011–2014 US National Health and Nutrition Examination Survey (NHANES): Test-retest reliability and validity testing. Chemosens. Percept..

[23] Murphy C (2002). Prevalence of olfactory impairment in older adults. JAMA.

[24] Sorokowski P, Karwowski M, Misiak M, Marczak MK, Dziekan M, Hummel T, Sorokowska A (2019). Sex differences in human olfaction: A meta-analysis. Front. Psychol..

[25] Preacher KJ, Hayes AF (2008). Asymptotic and resampling strategies for assessing and comparing indirect effects in multiple mediator models. Behav. Res. Methods.

[26] Benjamin DJ, Berger JO, Johannesson M, Nosek BA, Wagenmakers E-J, Berk R, Bollen KA, Brembs B, Brown L, Camerer C, Cesarini D, Chambers CD, Clyde M, Cook TD, De Boeck P, Dienes Z, Dreber A, Easwaran K, Efferson C, Fehr E, Fidler F, Field AP, Forster M, George EI, Gonzalez R, Goodman S, Green E, Green DP, Greenwald AG, Hadfield JD, Hedges LV, Held L, Hua Ho T, Hoijtink H, Hruschka DJ, Imai K, Imbens G, Ioannidis JPA, Jeon M, Jones JH, Kirchler M, Laibson D, List J, Little R, Lupia A, Machery E, Maxwell SE, McCarthy M, Moore DA, Morgan SL, Munafó M, Nakagawa S, Nyhan B, Parker TH, Pericchi L, Perugini M, Rouder J, Rousseau J, Savalei V, Schönbrodt FD, Sellke T, Sinclair B, Tingley D, Van Zandt T, Vazire S, Watts DJ, Winship C, Wolpert RL, Xie Y, Young C, Zinman J, Johnson VE (2018). Redefine statistical significance. Nat. Hum. Behav..

[27] Landis BN, Konnerth CG, Hummel T (2004). A study on the frequency of olfactory dysfunction: A study on the frequency of olfactory dysfunction. Laryngoscope.

[28] Bramerson A, Johansson L, Ek L, Nordin S, Bende M (2004). Prevalence of olfactory dysfunction: The Skövde population-based study. Laryngoscope.

[29] Vennemann MM, Hummel T, Berger K (2008). The association between smoking and smell and taste impairment in the general population. J. Neurol..

[30] Croy I, Nordin S, Hummel T (2014). Olfactory disorders and quality of life—An updated review. Chem. Senses.

[31] Doty RL (2017). Olfactory dysfunction in neurodegenerative diseases: Is there a common pathological substrate?. Lancet Neurol..

[32] Kondo K, Kikuta S, Ueha R, Suzukawa K, Yamasoba T (2020). Age-related olfactory dysfunction: Epidemiology, pathophysiology, and clinical management. Front. Aging Neurosci..

[33] Mackay-Sim A, John JS, Schwob JE, Doty RL (2015). Neurogenesis in the adult olfactory epithelium. Handbook of Olfaction and Gustation.

[34] Matulionis, D. H. Effects of the aging process on olfactory neuro plasticity. In *Olfaction and Endocrine Regulation: Proceedings of the Fourth European Chemoreception Research Organization Symposium and the Second International Laboratory Workshop on Olfaction at Schloss Lembeck, Near Essen, FRG, 11 to 15 October 1981*, 299–308 (IRL Press, 1982).

[35] McLeod M, Breen L, Hamilton DL, Philp A (2016). Live strong and prosper: The importance of skeletal muscle strength for healthy ageing. Biogerontology.

[36] Seubert J, Laukka EJ, Rizzuto D, Hummel T, Fratiglioni L, Bäckman L, Larsson M (2017). Prevalence and correlates of olfactory dysfunction in old age: A population-based study. J. Gerontol. Ser. A.

[37] Severinsen MCK, Pedersen BK (2020). Muscle–organ crosstalk: The emerging roles of myokines. Endocr. Rev..

[38] Doets EL, Kremer S (2016). The silver sensory experience—A review of senior consumers’ food perception, liking and intake. Food Qual. Prefer..

[39] Gopinath B, Russell J, Sue CM, Flood VM, Burlutsky G, Mitchell P (2016). Olfactory impairment in older adults is associated with poorer diet quality over 5 years. Eur. J. Nutr..

[40] Robinson SM, Reginster JY, Rizzoli R, Shaw SC, Kanis JA, Bautmans I, Bischoff-Ferrari H, Bruyère O, Cesari M, Dawson-Hughes B, Fielding RA, Kaufman JM, Landi F, Malafarina V, Rolland Y, van Loon LJ, Vellas B, Visser M, Cooper C, Al-Daghri N, Allepaerts S, Bauer J, Brandi ML, Cederholm T, Cherubini A, Cruz Jentoft A, Laviano A, Maggi S, McCloskey EV, Petermans J, Roubenoff R, Rueda R (2018). Does nutrition play a role in the prevention and management of sarcopenia?. Clin. Nutr..

[41] Doty, R. L. Epidemiology of smell and taste dysfunction. In *Handbook of Clinical Neurology* 3–13 (Elsevier, 2019) 10.1016/B978-0-444-63855-7.00001-0. 10.1016/B978-0-444-63855-7.00001-0 31604555

[42] Schubert CR, Cruickshanks KJ, Nondahl DM, Klein BEK, Klein R, Fischer ME (2013). Association of exercise with lower long-term risk of olfactory impairment in older adults. JAMA Otolaryngol. Head Neck Surg..

[43] Rosenfeldt AB, Dey T, Alberts JL (2016). Aerobic exercise preserves olfaction function in individuals with Parkinson’s disease. Parkinson’s Dis..

[44] Sharma JC, Vassallo M (2014). Prognostic significance of weight changes in Parkinson’s disease: The Park–weight phenotype. Neurodegener. Dis. Manag..

[45] Firth J, Stubbs B, Vancampfort D, Firth JA, Large M, Rosenbaum S, Hallgren M, Ward PB, Sarris J, Yung AR (2018). Grip strength is associated with cognitive performance in schizophrenia and the general population: A UK biobank study of 476559 participants. Schizophr. Bull..

[46] Marin C, Vilas D, Langdon C, Alobid I, López-Chacón M, Haehner A, Hummel T, Mullol J (2018). Olfactory dysfunction in neurodegenerative diseases. Curr. Allergy Asthma Rep..

[47] Camire M, Durán-Frontera E, Therrien M (2019). Risk factors for smell impairment and alteration in older Americans: NHANES 2013–2014 (P01-024-19). Curr. Dev. Nutr..

[48] Correia C, Lopez KJ, Wroblewski KE, Huisingh-Scheetz M, Kern DW, Chen RC, Schumm LP, Dale W, McClintock MK, Pinto JM (2016). Global sensory impairment in older adults in the United States. J. Am. Geriatr. Soc..

[49] Pinto JM, Wroblewski KE, Kern DW, Schumm LP, McClintock MK (2015). The rate of age-related olfactory decline among the general population of older US adults. GERONA.

[50] Devanand DP, Lee S, Manly J, Andrews H, Schupf N, Doty RL, Stern Y, Zahodne LB, Louis ED, Mayeux R (2015). Olfactory deficits predict cognitive decline and Alzheimer dementia in an urban community. Neurology.

